# An Accessible, Open-Source Dexterity Test: Evaluating the Grasping and Dexterous Manipulation Capabilities of Humans and Robots

**DOI:** 10.3389/frobt.2022.808154

**Published:** 2022-04-25

**Authors:** Nathan Elangovan, Che-Ming Chang, Geng Gao, Minas Liarokapis

**Affiliations:** New Dexterity Research Group, Department of Mechanical and Mechatronics Engineering, University of Auckland, Auckland, New Zealand

**Keywords:** dexterity test, grasping benchmarking, dexterous manipulation, dexterity, robot grasping, robot end effectors, cluttered scenes, grasping applications

## Abstract

Evaluating the dexterity of human and robotic hands through appropriate benchmarks, scores, and metrics is of paramount importance for determining how skillful humans are and for designing and developing new bioinspired or even biomimetic end-effectors (e.g., robotic grippers and hands). Dexterity tests have been used in industrial and medical settings to assess how dexterous the hands of workers and surgeons are as well as in robotic rehabilitation settings to determine the improvement or deterioration of the hand function after a stroke or a surgery. In robotics, having a comprehensive dexterity test can allow us to evaluate and compare grippers and hands irrespectively of their design characteristics. However, there is a lack of well defined metrics, benchmarks, and tests that quantify robot dexterity. Previous work has focused on a number of widely accepted functional tests that are used for the evaluation of manual dexterity and human hand function improvement post injury. Each of these tests focuses on a different set of specific tasks and objects. Deriving from these tests, this work proposes a new modular, affordable, accessible, open-source dexterity test for both humans and robots. This test evaluates the grasping and manipulation capabilities by combining the features and best practices of the aforementioned tests, as well as new task categories specifically designed to evaluate dexterous manipulation capabilities. The dexterity test and the accompanying benchmarks allow us to determine the overall hand function recovery and dexterity of robotic end-effectors with ease. More precisely, a dexterity score that ranges from 0 (simplistic, non-dexterous system) to 1 (human-like system) is calculated using the weighted sum of the accuracy and task execution speed subscores. It should also be noted that the dexterity of a robotic system can be evaluated assessing the efficiency of either the robotic hardware, or the robotic perception system, or both. The test and the benchmarks proposed in the study have been validated using extensive human and robot trials. The human trials have been used to determine the baseline scores for the evaluation system. The results show that the time required to complete the tasks reduces significantly with trials indicating a clear learning curve in mastering the dexterous manipulation capabilities associated with the imposed tasks. Finally, the time required to complete the tasks with restricted tactile feedback is significantly higher indicating its importance.

## 1 Introduction

Over the last decade a plethora of studies have focused on the development of dexterous robotic grippers and hands. However, the lack of a standardised definition and methods or tools for assessing and evaluating dexterity has resulted in researchers considering increased adherence to human-likeness to also offer increased dexterity, as discussed in (Liarokapis et al., 2013). This can be particularly attributed to the lack of appropriate dexterity metrics that properly define the various aspects of dexterity and quantify how dexterous specific robotic end-effectors are (Farrugia and Saliba, 2006). A tool or method for evaluating dexterity is of paramount necessity not only for designing new highly capable robotic end-effectors, but also for evaluating the skillfulness of humans in a variety of settings and application domains. Examples of such applications include, post injury rehabilitation assessment and standard skill assessment for specific professions (e.g., surgeons, pilots, construction workers etc.).

Human hand dexterity is generally defined as the ability of the hand to perform a desired motor task precisely and deftly with ease and skillfulness (Latash and Latash, 1994; Canning et al., 2000). Various functional evaluation tests have been employed by researchers over the years to assess and evaluate the dexterity of the human hand (Poirier, 1988). These tests can quantify the functional performance of human hands based on the ability of subjects to complete a wide range of tasks and industry specific tests. The outcomes of these dexterity tests can also serve as a valid indication of residual hand function after a severe injury or stroke in addition to being an evaluation of skillfulness. It is a common practice in industrial settings to use these dexterity tests for the purpose of screening and selection by evaluating the workers’ manual dexterity potential. The degree of improvement or the deterioration of hand functions during rehabilitation can also be determined by clinicians and researchers employing such tests (Chen et al., 2009). However, each of these tests is limited to a specific object range and task category. Also, most of these tests rely on stationary platforms and require the task to be completed in only one specific orientation. It is evident based on the analysis and discussion of the related work that there are a number of significant hand assessment tests that evaluate specific aspects of dexterity. However, there is a lack of comprehensive, holistic tests that evaluate dexterity as a whole and can be adapted to evaluate the capabilities of both robotic grippers and hands as well as human hands.

Robotic dexterity is generally being defined as the “*capability of changing the position and orientation of the manipulated object from a given reference configuration to a different one*” (Bicchi, 2000). The structure of robotic grippers vary widely from simple two fingered parallel-jaw grippers to highly complex anthropomorphic hands. Even within a class of grippers the design parameters vary widely. These variations have resulted in the lack of a common evaluation platform, benchmarks, metrics, and scores to evaluate the dexterity of robotic hands. Hence, there is a need for a dexterity assessment test that can evaluate the performance of robotic end-effectors, irrespectively of their design parameters. This need has also been identified in a roadmap that discusses the measurement science progress in quantifying robotic dexterity (Falco et al., 2014).

The various factors that contribute to robot dexterity are: *1*) the dexterity and skillfulness of robotic hardware components and *2*) the effectiveness of the perception and control system employed by the robotic system in the execution of dexterous tasks. The hardware component dexterity takes into account all the physical properties of the robotic gripper or hand, such as the mechanical design, the available degrees of freedom, the force exertion capabilities, the frictional properties etc., that contribute directly towards the grasping and manipulation performance of the system. The perception system on the other hand encompasses all the data that is captured and analyzed based on the information collected from the environment/surroundings of the robot, affecting the performance of the planning and control schemes of the robotic hardware system. In general, advanced sensing systems and complex control architectures have been deemed necessary for the execution of robust grasps and for the successful manipulation of a wide range of everyday life objects. However, a number of recent studies have demonstrated dexterous in-hand manipulation capabilities by employing underactuated, adaptive robot hands with minimal sensing and simple control schemes (Odhner and Dollar, 2015; Liarokapis and Dollar, 2016). The lack of commonly accepted methodologies to compare new algorithms and hardware across different robotic platforms is a topic of discussion in various workshops and forums organized by the robotic grasping and manipulation community (Quispe et al., 2018; IROS, 2020a). Our previous work involved developing a series of tests to evaluate dexterity of humans and robotic grippers on a static platform (Elangovan et al., 2020). We have expanded the work to include a dynamic environment and more complex manipulation tasks. In particular, in this work, we propose:

• A modular, accessible, open-source dexterity test that consists of a horizontal and vertical rig on which the manipulation tasks are to be performed. The rigs are mounted on a rotating module to simulate assembly task environments that require the tasks to be performed in varying orientations or in dynamic situations with varying obstacle spaces.
• A comprehensive set of tasks that evaluate the grasping and manipulation capabilities, and therefore the dexterity, of human and robotic hands. The proposed tasks range from simple pick and place to complex dexterous manipulation tasks.
• Evaluation protocols that provide quantitative dexterity metrics based on success rates and speed efficiency.
• A baseline score based on the analysis of human trials with and without tactile feedback.

The proposed dexterity test can serve as a valuable evaluation tool for determining the manual dexterity of human hands and for measuring the improvement in human hand function post injury. It can also evaluate the performance of robotic hands based on their ability to complete a task irrespectively of their individual design parameters, control systems, and sensing capabilities. The proposed dexterity test uses well defined measures of success (ability to complete a task successfully) and speed efficiency (time taken to perform a set of tasks) (Quispe et al., 2018), to calculate the overall performance of the human hand and robotic grippers.

The rest of the paper is organised as follows: Section 2 presents the related work that focuses on benchmarking dexterity, Section 3 presents the design of the dexterity test, Section 4 introduces the dexterity metrics used for the formulation of the benchmarking system, Section 5 discusses the validation of protocols and the baseline scores generated from human and robot experiments, while Section 6 concludes the paper and discusses some potential future directions.

## 2 Related Work

A plethora of dexterity tests have been proposed in literature to assess the dexterity and functionality of human hands (Yancosek and Howell, 2009). The development of such tests has helped in evaluation of manual dexterity and contribution of various hand anatomy attributes towards functional performance. These tests have also been adopted by studies focusing on the development of anthropomorphic hands to determine the degree of anthropomorphism and manual dexterity (Farrugia and Saliba, 2006). Each of these dexterity tests require the human hand to use various strategies for the successful grasping and manipulation of objects of specific shapes and sizes. The most commonly used assessment, the functional dexterity test (FDT) requires the hands to pick up cylindrical pegs placed in holes of a peg board and invert them. The ability of the user in performing a dynamic three-jaw chuck prehension is evaluated in (Aaron and Jansen, 2003). This is a common form of a pegboard test. Tests like the Purdue pegboard test, the Tweezers dexterity test, the Minnesota manual dexterity test among others are common variations of the peg board test (Buddenberg and Davis, 2000; Lundergan et al., 2007; Wang et al., 2011; Wang et al., 2018). All of these tests involve cylindrical pegs of various sizes ranging from small cylindrical pins (that need to be manipulated with tweezers) to huge cylindrical wooden pegs, to be picked, manipulated, and placed in holes on the peg board. These tests evaluate the speed and accuracy with which the hands being evaluated can pick, place, turn, and assemble. Another variation of the pegboard test, the grooved pegboard test requires the hands to place grooved metallic pegs into key holes that vary in orientations across the peg board (Wang et al., 2011). Hence, the hands need to re-orient the metallic pegs such that the pegs are aligned with the key holes prior to insertion.

The ASTM international has proposed a grasping dexterity test that evaluates a manipulator’s dexterity based on its ability to retrieve various blocks from a confined environment made up of an alcove composed of three shelves (Jacoff and Messina, 2007). The overall ability of the manipulator is determined based on the speed with which it can retrieve objects placed randomly in orientations that are not necessarily configured for the manipulator. Roeder manipulative aptitude test is another dexterity test that focuses on finger dexterity and speed in manipulating four fine components: rods, caps, bolts, and washers (Roeder, 1967; Çakıt et al., 2016). The first phase of the test involves screwing a rod onto the board followed by screwing a cap onto the rod within a fixed time period. The next phase of the test requires adding a bolt and washer alternatively to the T-bar mounted on the board. Furthermore, the hand-tool dexterity test can be used to evaluate the hands ability to use tools such as wrenches and screwdrivers (Bennett, 1965). The apparatus consists of two upright walls with one wall mounted with nuts, bolts, and washers. The time required by the subject to successfully move these components from the given wall to the other determines the tool dexterity score. Each of these tests is specific to particular object shapes and sizes and hence cannot be accepted as a generic dexterity score. Moreover, all these tests are performed on a stationary board/rig that has a fixed orientation throughout the evaluation. This is far from real world scenarios where the hands will need to adapt to wide range of orientations. Hence, in order to successfully evaluate the human hand function, tasks presented should require the hand to perform tasks in wide range of hand configurations or even in a dynamic environment with a dynamic obstacle space. These functionality evaluation tests have been adopted by studies focusing on anthropomorphic robots to quantify the dexterity of robotic grippers, comparing them with their human counterparts (Farrugia and Saliba, 2006; Saliba et al., 2013). A taxonomy of robotic manipulation benchmarks derived from the aforementioned studies has been proposed by Quisepe et al., classifying robot dexterity tests with three levels of increasing complexity: physical, dexterity, and functional tests (Quispe et al., 2018).

There are also certain evaluation tests developed exclusively for robotic dexterity evaluation that can be broadly classified into component benchmarking and system benchmarking. These tests require the robotic grippers to perform a set of manipulation tasks with a variety of objects under varying circumstances. As the name suggests, the component benchmarking focuses on specific components used for robotic grasping like the perception, control, mechanical hardware design etc. The system benchmarking on the other hand evaluates the capability of a complete robotic system as a whole to successfully execute tasks and has been the focus of a number of studies. Benchmarking studies pivot around the reproducability, adaptability, and scalability of the benchmarking environments and procedures to various platforms (Bonsignorio and Del Pobil, 2015). Hence, studies have focused on standardizing the testing platforms, objects, environments, and software. The YCB benchmarking system is considered one of the widely accepted benchmarking systems that facilitates replicable research by providing a varied set of standardised objects as well as associated models for evaluation and standardization of robotic end-effectors (Calli et al., 2015a). A number of studies have proposed benchmarking protocols that employ YCB object sets to evaluate the capabilities of robotic grippers and hands (Calli et al., 2015b; Yang et al., 2020).

A number of open-source simulation suites are available to compare the control strategies and learning algorithms irrespectively of their physical restrictions. Examples of such suites include the Gazebo, ALE (Arcade learning environment), openAI gym etc. They enable evaluation and comparison of various learning strategies and control algorithms independently of any physical restrictions (Koenig and Howard, 2004; Bellemare et al., 2013; Brockman et al., 2016). SURREAL is one such robotics suite that provides an accessible, open-source benchmarking tasks for reproducible manipulation research (Fan et al., 2018). Taking it further, PyRobot expands the open-source benchmarking system to include physical robots in addition to a Gazebo simulation suite thereby enabling the evaluation of hardware independent APIs (Murali et al., 2019). Some studies (ROBEL, REPLAB etc.) have also focused on developing a standardised hardware system for the evaluation of control strategies and learning algorithms irrespective of hardware limitations (Ahn et al., 2020; Yang et al., 2019).

In recent years, a number of robotic competitions have been organized with the intent to holistically evaluate different robotic platforms based on their ability to perform a set of tasks sequentially in a given, fixed environment. The DARPA robotic challenge (DRC) for instance evaluated effective manipulation as the key element in four of the eight tasks involved (DARPA, 2020). The Amazon Robotic Challenge (ARC) evaluates the capabilities of robotic grippers to pick and stock objects in a semi-structured environment representative of the shelves in an Amazon warehouse (Corbato et al., 2018). The benchmarking of robotic systems in manipulation and human-robot interaction in home environments is the key focus of the Robocup @ home competition that is organized by the Robocup initiative (ROBOCUP, 2020). The competition required the robots to manipulate objects of daily living positioned at predefined locations in the Robocup@home arena which is a realistic representation of an apartment setting consisting of various configurations like the kitchen, living room etc.(Matamoros et al., 2019). Similarly, a Robotic Grasping and Manipulation Competition has been organized continuously as part of the IEEE/RSJ International Conference on Intelligent Robots and Systems (IROS) since 2016 (IROS, 2019; IROS, 2020c). The competition focuses on the evaluation of the dexterity of robotic grippers and hands based on benchmarking tasks. These tasks are performed on a dedicated task board that incorporates four representative classes of industrial assembly tasks (IROS, 2020b).

A number of recent studies have proposed dexterity tests consisting of various object sets with the objective to evaluate and quantify specific aspects of dexterity. The features of these tests and how they compare with the test proposed in this paper are presented in the results section. Gonzalez et al., designed a Variable Dexterity Test (VDT) that consists of four subtests, each specifically designed to evaluate a particular type of grasp like the precision, cylinder, spherical, and extended spherical (Gonzalez et al., 2015). Another dexterity measurement kit proposed by (Saraf and Bisht, 2020) focuses on evaluation of pinch grasping capabilities of the fingers based on insertion, twisting, and locking tasks on a spring loaded wooden box. A simple and fast dexterity test for the evaluation of hand function called the peg test was presented by Noël et al. (2011). More recently a 3D printed platform that combined the features of multiple dexterity tests like the Box and Block test (BBT), Nine-Hole Peg test (NHP), and grooved peg board tests for the evaluation of fine manipulation and grasping capabilities has been proposed (Wilson et al., 2021).

Despite the plethora of studies focusing on benchmarking dexterity, there is a lack of commonly accepted evaluation systems across the robotics community. Given the increasing interests in the design and development of dexterous robotic grippers and hands, there is a need for a common benchmarking platform to quantify dexterity irrespectively of the design parameters. In our previous work, we had proposed an evaluation system that encompasses and builds upon important characteristics from the various commonly accepted dexterity evaluation methods reviewed (Elangovan et al., 2020). We further expand this work to include more complex manipulation tasks involving a dynamic rig that requires the object orientation to be changed constantly and new set of objects.

In particular, in this work, we propose a dexterity test that can evaluate the performance of a plethora of end-effectors solely on their task completion ability and speed of execution irrespectively of individual design parameters like number of fingers, actuators used, or control systems employed. Hence, it can be used to benchmark different classes and types of grippers and hands. For example, it can be used to evaluate the efficiency of devices such as suction grippers, parallel jaw grippers, two-fingered or three-fingered adaptive end-effectors, and anthropomorphic robot hands among others. The proposed dexterity test is equipped with both horizontal and vertical components that contain regions of specific manipulation tasks and objects that have been designed as described in section 3. A key aspect of this test is the ability to rotate, changing the position and orientation of the slots, requiring the hand to re-orient and re-position the objects in order to successfully complete the tasks. This simulates a dynamic assembly environment. The most important characteristic of the various benchmarking systems is a set of standardised objects that is representative of the set of objects encountered in industrial and home environments. However, most of the manipulated objects have been generally found to share similar characteristics (Matheus and Dollar, 2010). This fact has also been corroborated by Feix et al. that used video analysis of daily manipulation activities executed by household workers and machinists. Most of the objects manipulated by these workers had a weight of less than 500 g and required a grasp width of less than 70 mm (Feix et al., 2014). Deriving from these insights, a set of standardised objects have been proposed for evaluation as described in section 3.1. The types of objects used (sizes, shapes etc.) were chosen from state of the art dexterity tests that were proposed to evaluate specific aspects of manual/gross dexterity in rehabilitation and industrial settings. These tests provide insights on a subject’s ability to perform activities of daily living based on their performance in handling/manipulating simple objects like cylindrical pegs, cuboid blocks etc. For example, the Functional Dexterity Test (FDT) can assess the subjects capability in executing functional daily tasks involving any object that requires three-jaw chuck prehension based on a simple test performed with cylindrical pegs (Aaron and Jansen, 2003). Although the simple shapes of the objects may result in simple to secure grasps, the proposed tasks require the objects to be manipulated and assembled onto a rig that could also be moving. This requires the hands to re-orient the objects as they approach the rig. The designated holes for the examined objects have low tolerances during assembly. Thus, the complexity of task and the dexterity required for its execution are considerable. The complexity increases further during the execution of fine-manipulation tasks like fastening nuts onto bolts when they are in motion or performing thrust and twist motions to screw threaded pins into heat inserts etc., Assembly and disassembly of the puzzle tasks also require the hands to manipulate the outer covers of the puzzle by navigating them through a complex trajectory track on the inner block. Hence, this test can evaluate a wide range of manipulation capabilities using the simple set of objects proposed.

A set of standard operating procedures for task execution during the evaluation tests has been prepared so as to ensure the effectiveness of the benchmarking system. The sequence and conditions in which the test needs to be carried out, are presented in section 3.2 to ensure that the tests are organized under sufficiently similar conditions. Each set of experiment is repeated 3 times. The results of the three trials are used to examine the effect of familiarity to the tasks and the effect of mastering (over time) the manipulation capabilities. The evaluation method is described in section 4 and the scoring sheet is also made available. The proposed evaluation system is validated and baseline scores for the evaluation system are determined based on human evaluation trials and the results are presented in section 5.

## 3 Design of Dexterity Board

**FIGURE 1:**
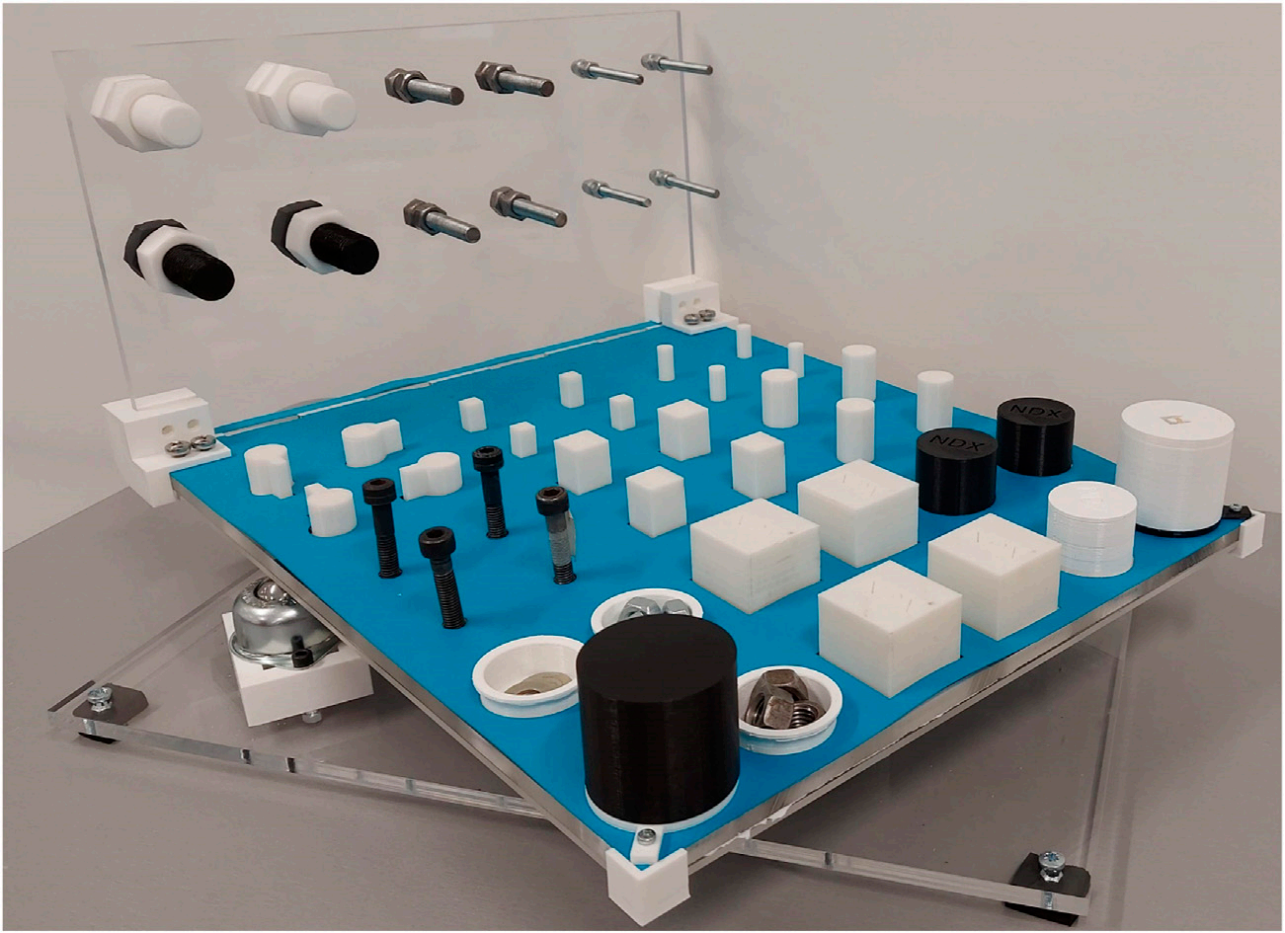
Prototype of the proposed dexterity test board that is equipped with a rotating base mechanism. The board is developed using plastic parts that are 3D printed and acrylic parts that are fabricated using laser cutting.

**FIGURE 2:**
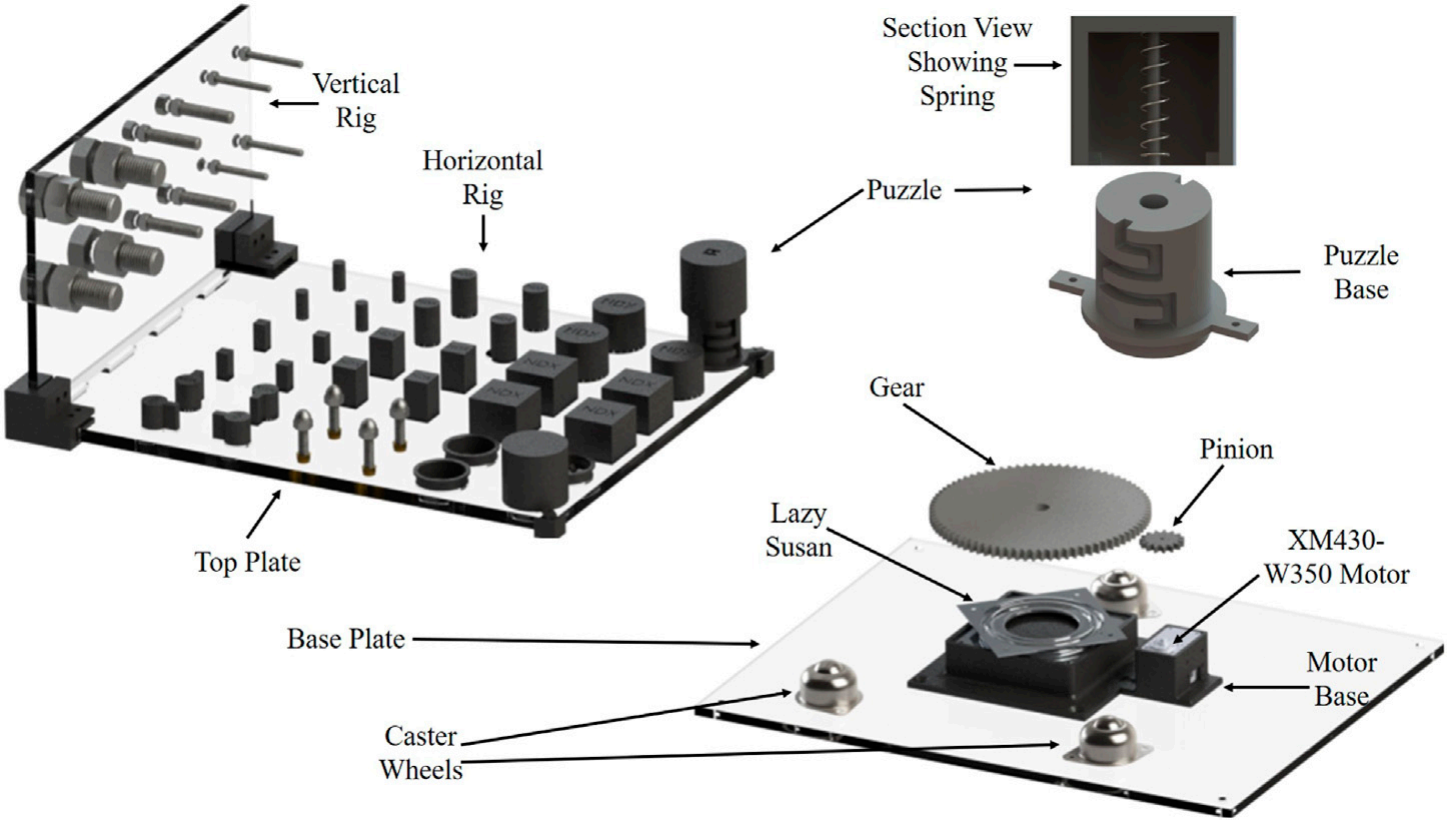
Exploded view of the proposed dexterity test board.

**FIGURE 3:**
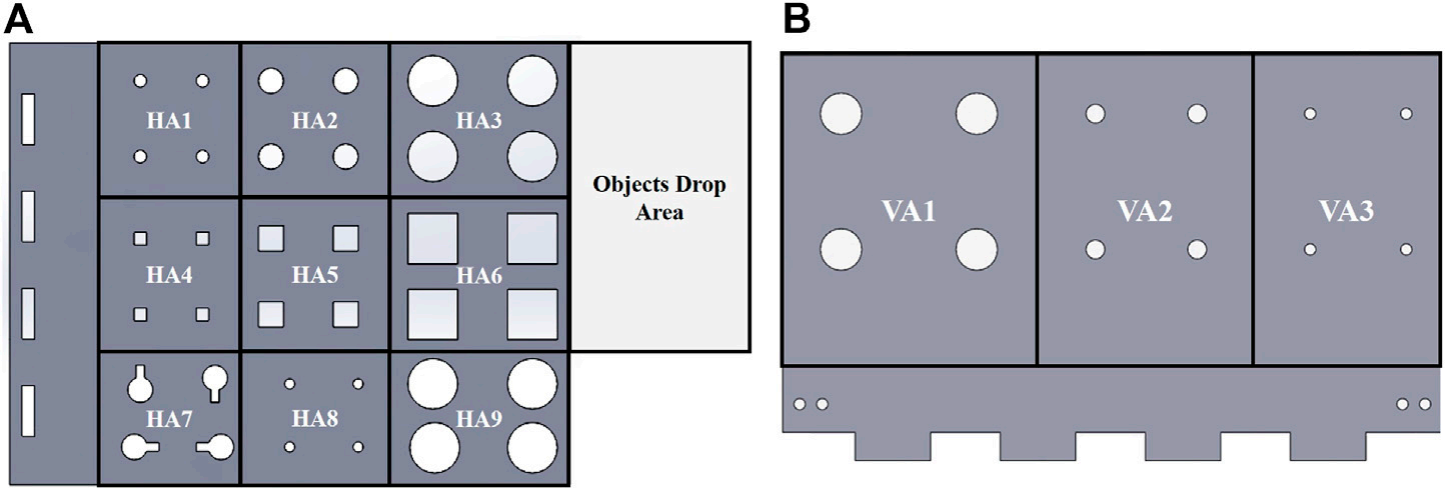
Manipulation regions/areas grouped based on the object being manipulated on: **(A)** the horizontal rig (HA1 - HA9) and **(B)** the vertical rig (VA1 - VA3).

The dexterity rig, as shown in Figure 1, is made up of a horizontal plate (450 × 350 mm) that is split into nine manipulation regions (HA1-HA9) and a vertical plate (350 × 200 mm) made up of three manipulation areas (VA1-VA3) shown in Figure 2. Each plate has a thickness of 10 mm. Each part of the regions is specific to a given set of objects and tests. Corner brackets are used to attach the vertical plate to the horizontal plate. The assembled test board is mounted onto a rotating base unit using a gear and lazy Susan mechanism that enables the entire test board to be rotated. The base mechanism is equipped with a Dynamixel XM430-W350 motor with a pinion gear mounted on it to drive the gear attached to the horizontal rig. This allows the test rig to be rotated at varying speeds in either clockwise or anti-clockwise direction. The base unit is fixed to a base plate that also has three inverted caster wheels supporting the horizontal rig plate, enabling smooth rotation of the rig. The exploded view of the proposed mechanism showing the various parts comprising the test, is shown in Figure 3. The various regions of manipulation on the horizontal and vertical plates are presented in Figure 2.

### 3.1 Objects

Custom 3D printed objects of varying shapes (cylinders, cuboids, and grooved pegs) and sizes have been designed for tests MT01 - MT13. The engraving on one face indicates the top side and is useful for benchmarking orientation. Tests MT14- MT22 employ standard threaded screws, washers, bolts, and nuts of three sizes (small, medium, and large), providing the range over which the robot hand needs to operate. Custom puzzles consisting of an inner and outer puzzle are designed for tests MT23 and MT24. The base of the inner puzzles can be screwed onto the horizontal plates in HA3 and HA9 regions respectively. A compression spring and an extension spring between the inner and outer puzzles are used to examine the capability of the gripper to exert sufficient forces during assembly and disassembly. Robot grippers can plan the grasping and manipulation strategies using the 3D models of the objects that are provided online. Table 1 summarizes the list of objects used, their dimensions, the specific manipulation region on the horizontal/vertical plate for the given task, as well as the task number, name, and detailed description.

**TABLE 1:** Dexterity test board components, regions, and task description grouped according to the five task categories and annotated with different colours.
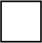
Pick and place
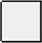
Re-orientation 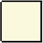
Fine manipulation
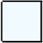
Tool task
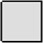
Puzzle manipulation

Objects		Object dimensions (mm)			Manipulation region			Task #	Task name			Task description		
Small Cylinder		10 × 28			HA1			MT01	Placing Task			Perceive, Grasp, Orient (Engraving on top), Position, Place		
								MT02	Turning Task			Grasp, Re-orient vertically (z-axis), Engraving on bottom, Place		
Medium Cylinder		20 × 38			HA2			MT03	Placing Task			Perceive, Grasp, Orient (Engraving on top), Position, Place		
								MT04	Turning Task			Grasp, Re-orient vertically (z-axis) Engraving on bottom, Place		
Large Cylinder		40 × 38			HA3			MT05	Placing Task			Perceive, Grasp, Orient (Engraving on top), Position, Place		
								MT06	Turning Task			Grasp, Re-orient vertically (z-axis) Engraving on bottom, Place		
Small Square		10 × 10 × 28			HA4			MT07	Placing Task			Perceive, Grasp, Orient (Engraving on top), Position, Place		
								MT08	Turning Task			Grasp, Re-orient vertically (z-axis) Engraving on bottom, Place		
Medium Square		20 × 20 × 38			HA5			MT09	Placing Task			Perceive, Grasp, Orient (Engraving on top), Position, Place		
								MT10	Turning Task			Grasp, Re-orient vertically (z-axis) Engraving on bottom, Place		
Large Square		40 × 40 × 38			HA6			MT11	Placing Task			Perceive, Grasp, Orient (Engraving on top), Position, Place		
								MT12	Turning Task			Grasp, Re-Orient vertically (z-axis), Engraving on bottom, Place		
Grooved Peg		10 × 28			HA7			MT13	Orienting Task			Perceive, Grasp, Orient (Engraving on top), Perceive, Re-orient (Key groove aligned to key hole), Place		
Threaded Pins		M8 1.25 mm thread, 40 mm			HA8			MT14	Thrust and Twist			Perceive, Fine grasp, Orient, Position, Place, Thrust and Twist		
								MT15	Twist and Pull			Perceive, Fine grasp, Twist and Pull, Position and Place		
Washers and Nuts		M6 or higher			VA3			MT16	Insertion Task			Perceive, Fine grasp, Orient (Concentric to M6 screw), Position, Place		
M6 Bolt		M6 1 mm thread, 60 mm			VA3			MT17	Tool Task (Assemble)			Grasp nut (robustly), Orient nut to bolt tip, Manipulate, Re-orient, Manipulate		
					VA3			MT8	Tool Task (Disassemble)			Grasp nut (robustly), Orient, Manipulate, Re-orient, Manipulate		
M10 Bolt		M10 1.5 mm thread, 60 mm			VA2			MT19	Tool Task (Assemble)			Grasp nut (robustly), Orient nut to bolt tip, Manipulate, Re-orient, Manipulate		
					VA2			MT20	Tool Task (Disassemble)			Grasp nut (robustly), Orient, Manipulate, Re-orient, Manipulate		
M22 Bolt		M22 2.5 mm thread, 60 mm			VA1			MT21	Tool Task (Assemble)			Grasp nut (robustly), Orient nut to bolt tip, Manipulate, Re-orient, Manipulate		
					VA1			MT22	Tool Task (Disassemble)			Grasp tool (robustly), Orient tool tip to nut, Manipulate, Re-orient, Manipulate		
Puzzle 1		Puzzle with Compression Spring			HA9			MT23	Disassemble and Assemble			Grasp puzzle, Rotate left/right, Lift (Disassemble), Rotate left/right and Push down (Assemble)		
Puzzle 2		Puzzle With Extension Spring			HA3			MT24	Disassemble and Assemble			Grasp puzzle, Rotate left/right, Lift (Disassemble), Rotate left/right and Push down (Assemble)		

### 3.2 Manipulation Tasks

Twenty four benchmarking tasks have been broadly classified into five manipulation categories. These tasks are numbered MT01-MT24.

These tasks have been adapted from existing dexterity tests as well as challenges designed to provide an insight of the hand efficiency in assembly, packing, tool and machine operation, and other jobs. The tasks are as follows:

• Simple Manipulation Tasks (MT01, MT03, MT05, MT07, MT09, MT11): The initial positions and orientations of the objects to be manipulated in both industrial settings and home environments are generally randomized. To render the testing conditions similar to this, cylindrical and cuboidal objects of varying sizes are cluttered in random initial orientation within the reachable workspace of the robot. The robot gripper or hand is then needs to perceive, pick these objects from a random initial pose, position them, and place them in designated holes on the horizontal rig with a specific orientation. Successful execution of these tasks evaluates the gripper’s perception capability to identify the initial position and orientation of the objects, planning the best approach to grasp, orient the objects such that the engraving is on top, and sequentially place them into respective holes.
• Re-orientation Tasks (MT02, MT04, MT06, MT08, MT10, MT12, MT13): One of the key manipulative skill of the human hand lies in its ability to re-orient objects along one or more axes within its workspace. The tasks in this category examine the capability of the end-effector to grasp an object from any given orientation, rotate the object along one or more axes, and place the object in designated holes in a very specific final orientation. The robot’s ability to successfully complete the tasks serves as a direct indicator of its perception of the position and orientation of object and target hole during the reach to assemble phase, as they must be aligned before the execution of the insertion task. Cylindrical and cuboidal objects need to be inverted for tasks MT02, MT04, MT06, MT08, MT10, and MT12 while MT13 requires the reorientation and placement of grooved pegs into key shaped holes.
• Fine Manipulation Tasks (Fine Component Manipulation Tasks - Set A) (MT14, MT15, MT16): The tasks in these category evaluate fine manipulation capabilities of fingers and hands required for assembly and disassembly of fine components such as washers and nuts. These tasks evaluate the gripper’s ability to pick up small components (like nuts, washers), orient them, and screw/fasten them onto other components to create an assembly. Fine finger movements like thrust and twist, and twist and pull motions are evaluated in tests MT14 and MT15. The threaded pins are placed in random initial orientation in the object drop area. The gripper needs to grasp one threaded pin at a time, orient them onto the designated holes with heat inserts on the horizontal region (HA8), thrust and twist to screw the pins in. The next task requires unscrewing the threaded pins one at a time by twist and pull motions and place them back in the object drop area. The final set of tasks in this category require the gripper to grasp small components (washers and nuts) placed in small trays in the HA9 region of the board and insert them alternatively onto a screw mounted in the VA3 region of the vertical plate.
• Tool Tasks (Fine Component Manipulation Tasks - Set B) (MT17 - MT22): These tasks are an extension of the arm-hand manipulation tasks described above and require finer control of small components to be completed. The tasks in this category evaluate the dexterity associated with picking, precision placement, assembling, disassembling, and fitting together parts without any tools. These are complex tasks that require the end-effector to robustly grasp fine components such as nuts of varying sizes (small, medium, and large), place them precisely onto tips of screws that are mounted on the vertical rig, and tighten them onto the screws as the rig is rotating. This is followed by disassembling the nuts from the screws and placing them back in the trays located in the HA9 region. The components will have to be grasped robustly, and re-oriented multiple times for the successful completion of the task as the rotation of the rig causes the orientation of the screws to vary continuously. The complexity of tasks in this category require a high level of dexterity for successful task execution. Hence, the rate of success and completion time for this task category can serve as a valid indicator of the gripper dexterity.
• Puzzle Manipulation Tasks (MT23 - MT24): These tasks employ two cylindrical puzzles fixed onto horizontal regions HA3 and HA9. Each puzzle is made up of an inner and outer puzzle component attached to each other with a compression spring (puzzle 1) and extension spring (puzzle 2). Successful completion of the tasks require the outer component to be grasped and navigated through the puzzle engraved on the inner component by manipulating it clockwise and anti-clockwise, and lifting it all the way up until each puzzle is completely disassembled. This needs to be followed by assembling the puzzle back by guiding the outer component through the puzzle route on the inner component until the puzzle is completely assembled.

## 4 Dexterity Metrics

In this section, we introduce metrics based on the successful task completion (ability) and rate of completion (speed) of the tasks. The total score is then presented as a weighted average of these individual scores. The final part of this section presents a ranking and grading system that would allow easy comparison of grippers and choose the ideal gripper for a particular set of tasks. The metrics are as follows.

### 4.1 Successful Completion Score

Each of the tasks described in section 3.2 is repeated with four objects sequentially and a point is awarded for each successful completion. Hence, the score for any given task “i” can vary between “0” to “4.” And Eq. 1 describes *S*_*s*_ (Successful completion Score), the ability of the gripper to successfully complete all the tasks.

$$S_{s}=\frac{1}{P_{\max}}\;\overset{n}{\underset{i=1}{\sum }}P_{i},$$ (1)

Where *n* is the total number of tasks (24) and the term *P*_max_ denotes the maximum possible score that can be achieved by a gripper completing all the *n* tasks and can be written as *P*_max_ = 4*n*. *P*_*i*_ is the number of objects successfully manipulated for the *i* ^*th*^ set of task and can vary between “0” and “4.” The total points achieved by a gripper $\sum_{i=1}^{n}P_{i}$ denotes the total score of successful completion and can be replaced by the term as *P*_*total*_. Eq. 1 can now be rewritten as,

$$S_{s}=\frac{P_{total}}{P_{\max}},$$ (2)

Equation 2 provides us with a Successful completion score *S*_*s*_ that varies between 0 and 1. The lower end of the scale represents a non-dexterous device incapable of executing any tasks and the higher end of the scale represents a highly dexterous device capable of successfully executing all the manipulation tasks.

### 4.2 Time Required Score

The metrics introduced in this section can be used to measure the rate of task completion. Task completion time can vary between each individual task depending on the objects, the initial orientation of the objects, grasp planning, approach, and manipulation strategy employed. Equation 3 provides *S*_*t*_ (Time required score), the speed with which the gripper can complete the tasks.

$$S_{t}=\frac{\log\left(T_{\min}\right)}{\log\left(\overset{n}{\underset{i=1}{\sum }}T_{i}\right)},$$ (3)

Where *T*_min_ is the minimum time taken for task completion obtained from the human experiments. We consider human performance as the baseline. The time required for completion of *i* ^*th*^ task is given by *T*_*i*_. The cumulative time taken for all *n* tasks is calculated as $\sum_{i=1}^{n}T_{i}$ and can be written as *T*_*total*_. Equation 3 can now be rewritten as,

$$S_{t}=\frac{\log\left(T_{\min}\right)}{\log\left(T_{total}\right)},$$ (4)

Equation 4 provides us with a time required for task completion score *S*_*t*_ that varies between 0 and 1. A higher time score *S*_*t*_ indicates the ability of the gripper to complete the manipulation tasks at a faster rate and thus signifies better dexterity.

### 4.3 Total Dexterity Score

The total dexterity score *S*_*total*_ is calculated from the weighted sum of successful completion score (*S*_*s*_) and time required score (*S*_*t*_). This metric provides us with the combination of grippers ability and speed in completing the various manipulation tasks. To allow for easy comparison, the total score is presented on a percentage scale ranging from 0 to 100% as shown in Eq. 5.

$$S_{total}=\left(w_{s}S_{s}\;+\;w_{t}S_{t}\right)\;*\;100,$$ (5)

Replacing the values of *S*_*s*_ and *S*_*t*_, the equation can be rewritten as,

$$S_{total}=\left(w_{s}\left(\frac{P_{total}}{P_{\max}}\right)\;+\;w_{t}\left(\frac{\log\left(T_{\min}\right)}{\log\left(T_{total}\right)}\right)\right)\;*\;100,$$ (6)

The weight constants for successful completion (*w*_*s*_) and time (*w*_*t*_) are used to vary the importance of individual sub-score. The sum of these constants must be equal to 1 (*w*_*s*_ + *w*_*t*_ = 1). If the weights are assigned an equal value (0.5 each) the equation would distribute equal importance to the ability and speed of task completion. In case of evaluating the grippers ability to perform certain complex tasks irrespective of time taken to complete them, a greater value could be assigned to *w*_*s*_. The results are presented on a scale ranging from 0 (simplistic, non-dexterous system) to 1 (human-like, dexterous system). This score represents the capabilities of a gripper or hand to perform complex grasping and manipulation tasks compared with the human hand, which is considered to be Nature’s most effective and dexterous end-effector. If a gripper can perform all the tasks successfully within the baseline time determined by human experiments, the gripper is considered to be highly dexterous exhibiting human-like grasping and manipulation performance.

### 4.4 Dexterity Ranks and Grades

In order to classify and compare the robot grippers amongst each other, a system of ranks and grades is introduced. This grading system helps decide on the ideal gripper for a given set of tasks. The ranks for the robot grippers can vary from “0 star” to “5 stars” (one star corresponding to each task category). A robot gripper is awarded one star on successful completion of all the tasks in a given task category. No star is awarded if the gripper fails in executing any of the tasks. Hence, a robot’s dexterity can be easily verified based on the number of stars from “0 stars” (non-dexterous) to “5 stars” (most dexterous). For example, if a hand can accomplish all the tasks in three of the five task categories, its rank would be “3” stars. To differentiate between hands that are equally ranked, a grading system consisting of six grades is provided. If none of the tasks in a task category can be executed, it is graded as “F” and an “A” is awarded for grippers capable of completing all the tasks successfully. The detailed grading system is presented in Table 2. This ranking and grading system serves as an indicator of the robot’s overall performance as well as its individual capabilities in successful execution of various task categories. If the requirement is for a simple pick and place tasks, an “1” star robotic gripper that has graded “A” for pick and place task would be well suited rather than a complex “5” starred gripper. Thus, this ranking and grading system shall help identify grippers suitable for various needs and task categories.

**TABLE 2:** Grading system for the grippers based on successful task completion in a given task category.

Tasks completed	Grades
No tasks completed	F
Tasks <1/3 (*T*_*total*_)	D
1/3 (*T*_*total*_)<Tasks <2/3 (*T*_*total*_)	C
Tasks >2/3 (*T*_*total*_)	B
All tasks completed	A

## 5 Validation of Protocols and Baseline Score of Human Trials

**FIGURE 4:**
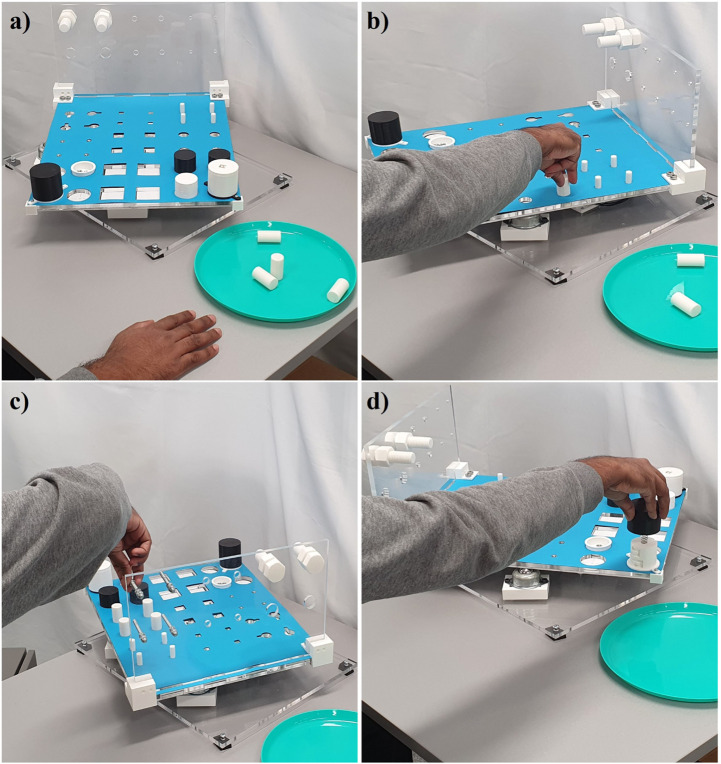
A subject performing experiments executing tasks of the dexterity test. The subfigures show: **(A)** the initial position of the hand and objects, **(B)** a placing task, **(C)** a tool task, **(D)** a puzzle task. As shown in the images the orientation of the dexterity board constantly changes requiring the arm-hand system to adapt to various orientations to complete the tasks successfully.

The benchmarking protocols detailed in section 3 were executed by humans to validate their efficiency and the results were calculated using Eqs 1, 3, 6 to obtain a dexterity score. The average of these human hand experimental results is used as a baseline score for comparison and evaluation of human hand dexterity, as well for comparing the dexterity of other robotic grippers and hands. This study recruited ten healthy subjects whose arm lengths were 76.15 ± 4.48 cm. The University of Auckland Human Participants Ethics Committee approved this study study (reference number #019043), and all participants gave informed consent. The subjects sat in a comfortable position for the entire duration of the experiments, with the forearm placed to the right of the dexterity test as an initial configuration. Three sets of experiments were performed by the subjects as shown in Figure 4. Each subject repeated the tasks MT01 - MT24 sequentially for three trials for the first set of experiments without gloves. The experiments were then repeated with a padded palm, high grip glove for three trials to determine the effect of reduced tactile sensing on dexterous manipulation capabilities. For both the sets of experiments, the dexterity rig was rotating at a constant speed of 3 RPM to examine the subjects ability to adapt to a dynamic test environment in terms of perception, planning, and manipulation capability. A third set of experiments involved performing the tasks on a stationary rig for three trials in order to determine the effect of static against dynamic environments on the performance of the participants. The detailed evaluation protocol with explanatory images and scoring sheets, as well as the open-source CAD files of the proposed dexterity test are provided and can be downloaded from the following website: http://www.newdexterity.org/dexteritytest 

The particular website will also serve as a repository of the scores and evaluations of various robotic hands and grippers.

### 5.1 Results and Discussion

**FIGURE 5:**
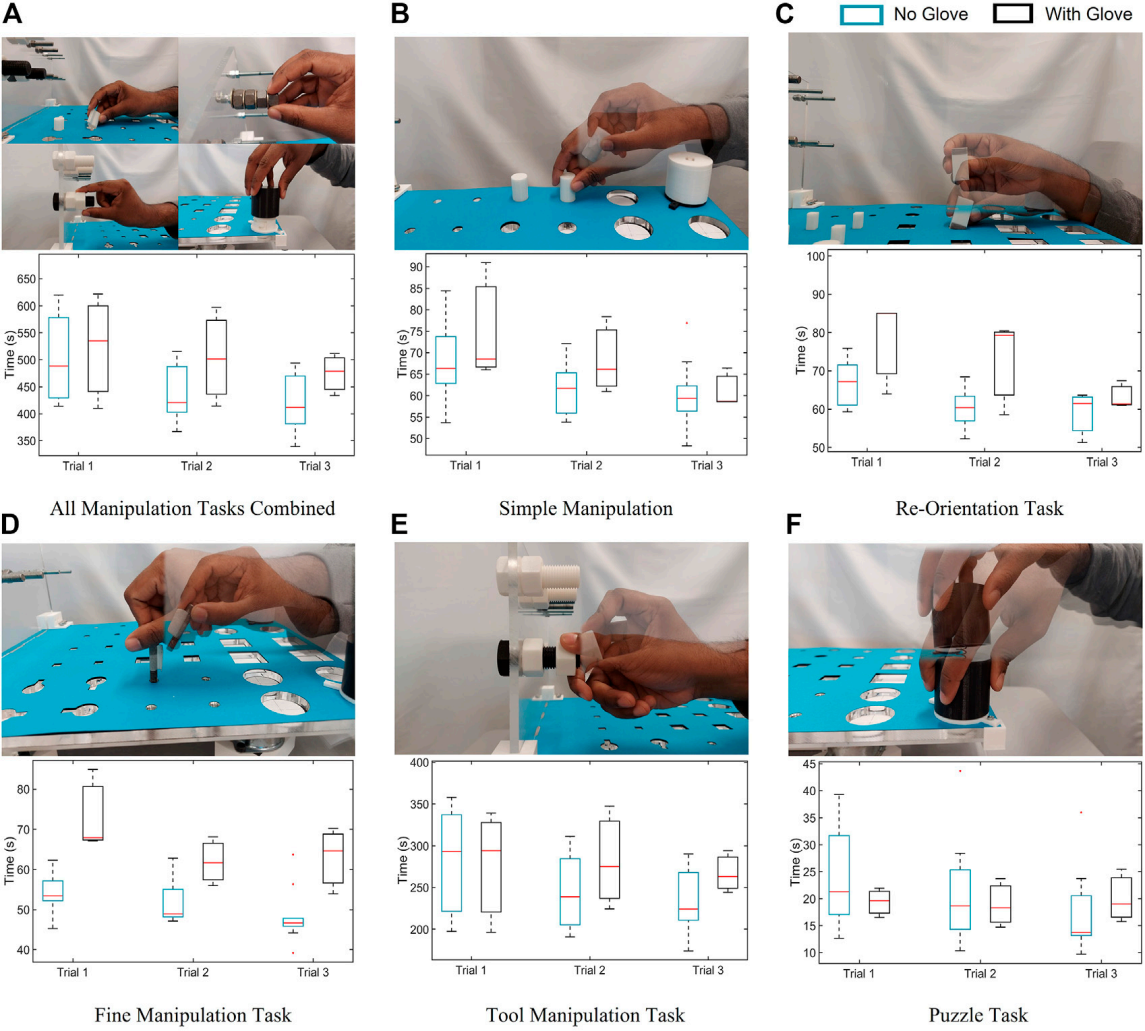
The time taken by 10 subjects to complete various manipulation tasks across three trials with and without gloves is presented for: **(A)** all tasks combined, **(B)** simple manipulation tasks, **(C)** re-orientation tasks, **(D)** fine manipulation tasks, **(E)** tool manipulation tasks, and **(F)** puzzle tasks. The figure also presents a visual representation of the tasks from each of the five different task category.

The various features being evaluated in this study and how they compare with other existing dexterity tests is shown in Table 3. Figure 5 presents a visual representation of the tasks and the results of the experimental trials corresponding to each of the five different task category. In order to determine the degree of variation, we calculated the percentage co-efficient of variation (%CV) for each of the completion time for each given task category and the overall completion time using

$$\%CV_{t}=\frac{\sigma_{t}}{\mu_{t}}*100,$$ (7)

Where, *σ*_*t*_ and *μ*_*t*_ are the standard deviation and mean for a given task category *t*. The *%CV*_*t*_ for the overall completion time for all the participants across three trials was 13%. The %CV for all the individual task categories were less than 20%. These values of %CV signify low dispersion time across various subjects and trials and helps in validating the efficiency of the experimental protocols across various subjects. Hence, the values derived from these experiments could be used as baseline score for human and robot dexterity evaluation experiments. There was no significant correlation between the arm length of the subjects and the performance for the task categories examined in this study. It can also be noted from the plots that the average time taken by the subjects to complete the tests in each of the individual task category was significantly lower than the time taken in the previous trial. The results are validated using ANOVA to determine the statistical significance between the trials. A *p*-value of 0.013 (less than the alpha value of significance, 0.05) for the total time taken to complete the tasks across the three trials indicates that the time taken for successive trials decreases significantly. This could be attributed to the subjects familiarity to the tasks and hence indicates that dexterity improves with repetition. Dexterity could then be considered a learned attribute that can be improved by exercising specific sets of tasks.

**TABLE 3:** Table comparing the test environment, object sets, and features being evaluated across various dexterity tests proposed in recent literature.

Studies	Designed for		Test environment	Object set	Task categories					Obstacle
	Humans	Robots			PP	RO	FM	TT	PT	
Noël et al. (2011)	*✓*		vertical planes	Plastic pegs	*✓*					N/A
Gonzalez et al. (2015)	*✓*		Two wooden Boards	Custom made plastic objects (four sets)	*✓*					N/A
DARPA (2015)		*✓*	Four sequential courses	Manipulation tasks of varying complexity (four tasks)		*✓*	*✓*			Stationary
ARC (2016), Corbato et al. (2018)		*✓*	Shelving unit structured in 12 bins	Objects Representative of objects handled in amazon warehouse (39 objects)	*✓*	*✓*				Stationary
Saraf and Bisht (2020)	*✓*		Single holed, spring loaded wooden box	3D printed Pegs	*✓*	*✓*	*✓*			N/A
IROS (2020d)		*✓*	Dedicated taskboard	Objects Representing different classes of Industrial assembly (four sets)	*✓*	*✓*	*✓*	*✓*		Stationary
ROBOCUP (2020)		*✓*	Home Environment arena with structured rooms	Categorized Objects (30 objects)	*✓*	*✓*				Stationary
Wilson et al. (2021)	*✓*		3D printed Platform	3D printed pegs	*✓*	*✓*				Stationary
This study	*✓*	*✓*	Dynamic board	3D printable Objects and standard bolts/nuts (14 sets)	*✓*	*✓*	*✓*	*✓*	*✓*	Dynamic

The task categories are abbreviated as PP, Pick and Place, RO, Re-Orientation, FM, Fine Manipulation, TT, Tool Task, PM, Puzzle Manipulation.

**FIGURE 6:**
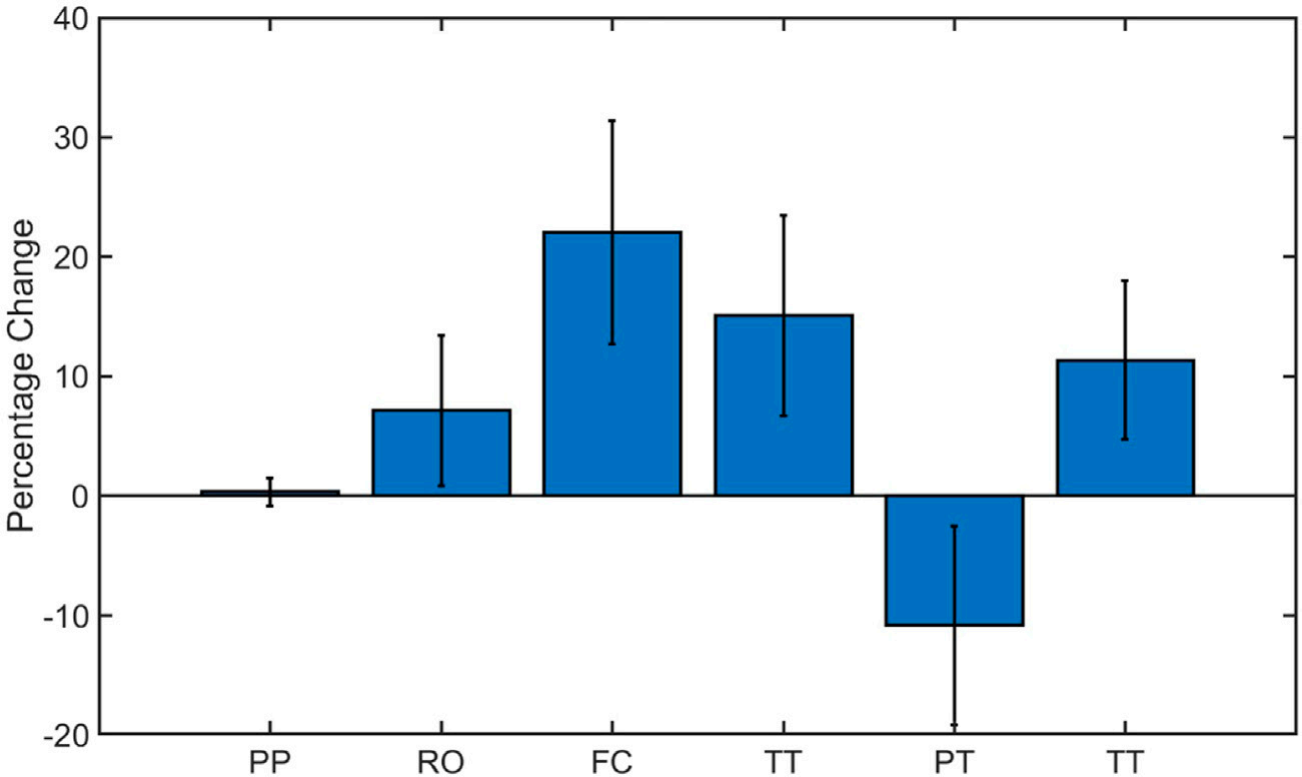
Percentage change in manipulation time with gloves on for the various task category: Pick and Place (PP), Reorientation (RO), Fine Component Manipulation (FC), Tool Task (TT), Puzzle Task (PT) and Total Time (TT).

In order to determine the effect of tactile sensing on the dexterous manipulation capabilities, the experiments were repeated with gloves on and the results are presented in Figure 5. This had varying effects for various task categories, as shown in Figure 6. The time taken to complete the pick and place task category was identical with the gloves on and off. The effect was most pronounced for fine manipulation tasks where the average completion time was higher by 30% when performed with the gloves. On the other hand, the puzzles could be solved 12% faster with the gloves on. Similar to the first set of experiments, the tasks were completed faster when the trials were repeated indicating the learning curve is effective in improving dexterous manipulation even with the gloves on. ANOVA resulted in a *p*-value of 0.0066 which is less than the alpha value of significance (0.05) and one would reject the null hypothesis, as there is strong evidence that the values between trials differ. The percentage co-efficient of variation (%CV) for all the individual tasks as well as the combined total time was under 20%. The results obtained from these two set of human experiments is presented in Table 4. These results serve as the baseline scores for the dexterity tests.

**TABLE 4:** Table presenting the baseline scores for the dexterity tests performed by a human with a glove and without a glove.

Task category	No glove			With glove		
	Average Time(s)	Standard deviation	Coefficient of variance	Average time (s)	Standard deviation	Coeffecient of variance
Total Time	452.10	57.65	12.67	500.31	64.46	12.72
Pick and Place	63.49	7.04	11.07	68.28	7.41	10.54
Reorientation	62.28	5.05	8.08	71.33	7.64	10.39
Fine Manipulation	51.20	5.61	7.55	66.04	5.84	6.24
Tool	253.51	44.76	17.52	275.21	43.55	15.72
Puzzle	20.72	4.55	21.95	19.44	3.31	14.71

**FIGURE 7:**
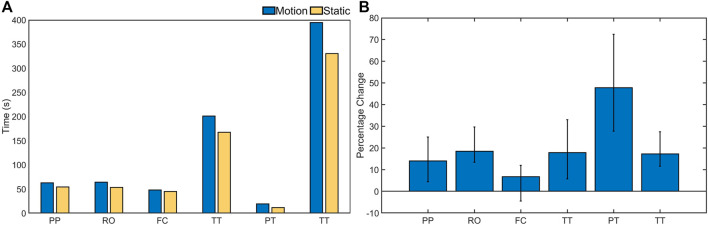
Subfigure **(A)** presents a comparison of time taken by the subjects to complete various task category in seconds when the rig was static and in motion (rotating). Subfigure **(B)** presents the percentage increase in completion time for the various task category when the rig was in motion. The task categories are: Pick and Place (PP), Re-Orientation (RO), Fine Component Manipulation (FC), Tool Task (TT), Puzzle Task (PT), and Total Time (TT).

**FIGURE 8:**
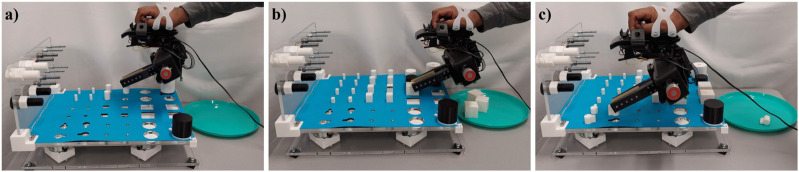
A subject performing the experiments on the dexterity test board with a palm mounted interface to control a Multi Modal Parallel jaw gripper, performing: **(A)** a placing task of a medium cylinder, **(B)** a placing task of a large cylinder, **(C)** a re-orientation task of a grooved peg.

**FIGURE 9:**
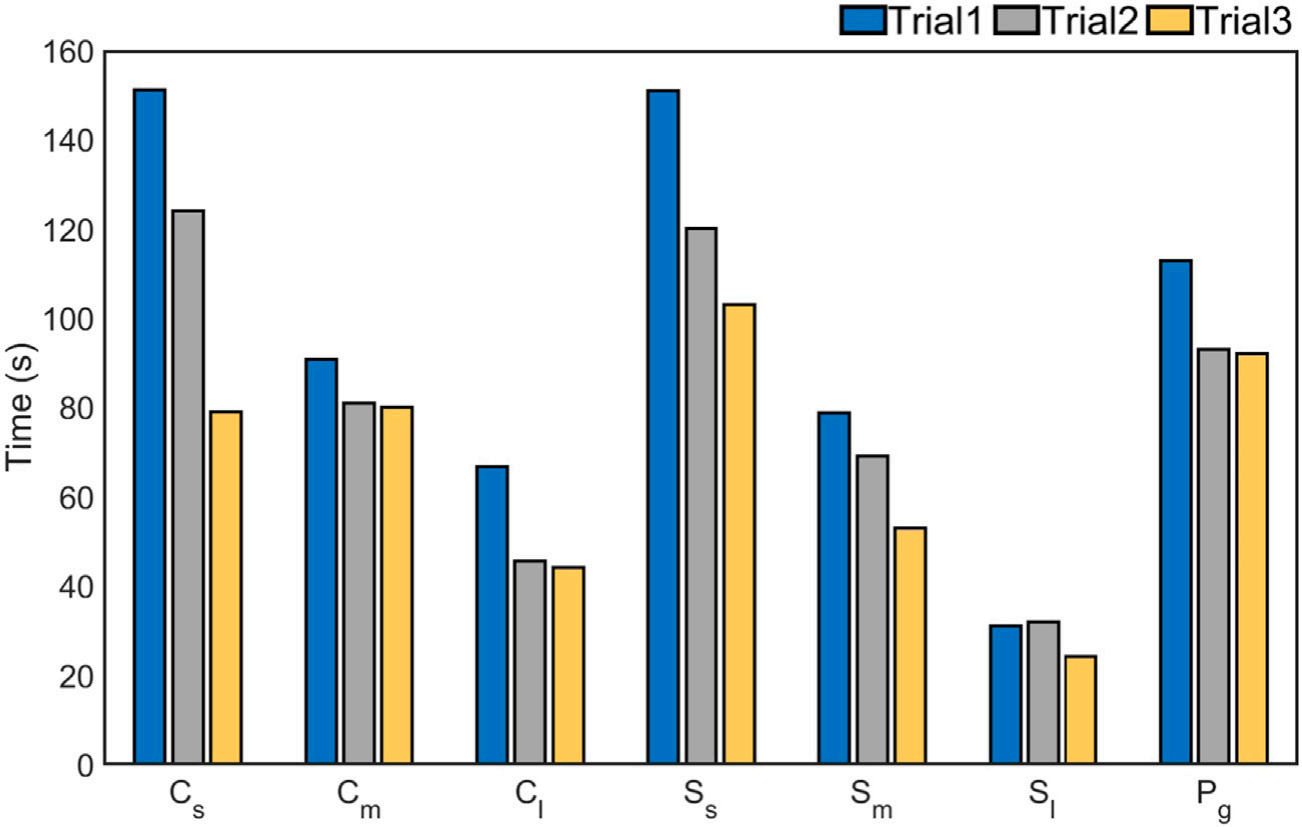
The comparison of time taken by the Multi Modal Parallel jaw gripper to complete tasks for objects of varying shapes and sizes, is presented. C, S, and P stand for Cylinders, Squares and Pegs respectively. The subscripts s, m, l, g, denote small, medium, large, and grooved parts respectively.

**FIGURE 10:**
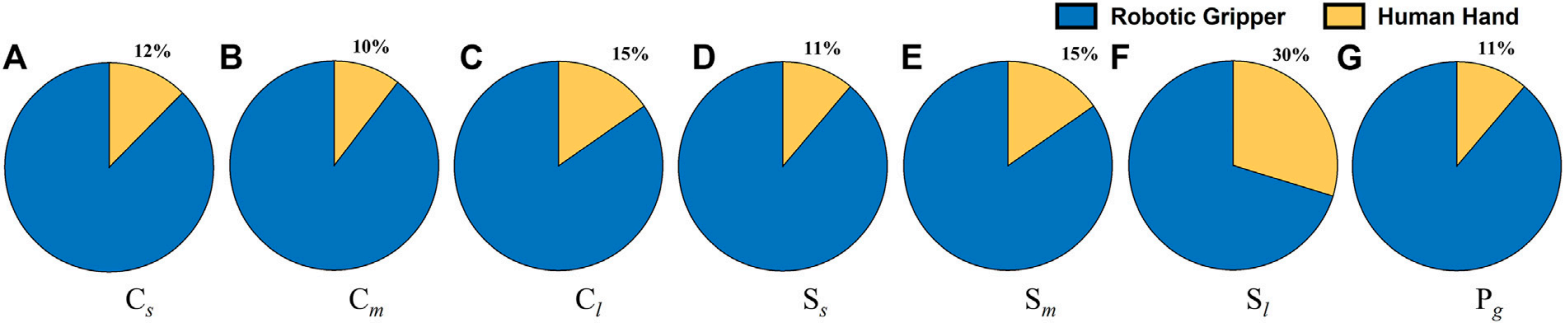
This pie chart presents a comparison of the time taken by the robotic grippers against human hands for executing tasks with objects of varying shapes and sizes. C, S and P stand for Cylinders, Squares and Pegs respectively. The Subscripts s, m, l, g denote small, medium, large, and grooved objects respectively.

Further, a third set of experiments was performed for three trials on a stationary rig to investigate how much dexterous manipulation capability improved on a fixed rig as opposed to when one in motion was used. The co-efficient of variation for all the task categories was well under 13% indicating a closer dispersion time across subjects in completing the tasks on a stationary rig. The result of this experiment is presented in Figure 7. It is clear from the plots that the task completion time was faster on a stationary rig for all the task categories. This effect was most prominent for the puzzle task which took 40% more time when the rig was moving. As opposed to this the fine manipulation task category was slower only by 7%. As with the previous set of experiments, the task completion time decreased across the trials further confirming the effects of learning curve on the execution of dexterous manipulation tasks. The experiments were also performed with a multi modal parallel gripper mounted on a palm interface as shown in Figure 8 to determine the dexterous manipulation capabilities of the gripper and to investigate the effect of learning on manipulation capabilities across trials. The gripper was unable to complete all the task categories as it lacked the complex in-hand manipulation capabilities required for successful task completion in these categories. However, task completion time reduced significantly with each consecutive trial for all the tasks that could be successfully completed, as presented in Figure 9. This further supports the argument that dexterity can be learned and improved by performing a particular set of tasks repeatedly. Figure 10 presents the pie chart comparing the percentage of time taken by human hand to complete the various tasks against the time taken by the robotic gripper. As seen from the pie chart, the human hands can complete the tasks in a very small fraction of the time taken by the robotic grippers. This shows that there is a huge room for improvement of robotic devices.

## 6 Conclusion

In this paper, we proposed a new modular, affordable, accessible open-source dexterity test that evaluates the grasping and manipulation capabilities of humans and robotic hands and other end effectors by combining the features of multiple human dexterity tests as well as new task categories specifically designed for robots. These tests help quantify the manual dexterity of humans apart from evaluating the human hand function improvement post injury. The features from many existing hand function tests, along with new features such as the rotating module and the dexterity puzzles make this test one of the most comprehensive dexterity evaluation systems. Apart from this, the test also involves benchmarking tasks that evaluate key robotic manipulation capabilities identified from literature and robotic challenges. A set of dexterity metrics have also been proposed that quantify the dexterity of robot grippers and hands by evaluating their ability to complete these tasks on a scale ranging from 0 (simplistic, non-dexterous system) to 1 (human-like, dexterous system). The scores are based on the hands’ ability to complete the tasks successfully with accuracy and precision, as well as the speed at which the tasks can be executed. The weighted sum of the successful completion and speed of completion is used to obtain the final dexterity score. Further alternative measures in the form of dexterity ranks and grades enable comparison of various grippers and their manipulation capability in an intuitive manner irrespectively of their individual design parameters. Thus, the proposed dexterity test and metrics provide researchers around the world with benchmarking methods and tools that can be easily replicated to quantify the ability of robotic end-effectors to perform complex tasks effectively, allowing the comparison of their grippers against various classes of grippers. The accompanying website shall serve as an open access repository of dexterity scores for robot hands and grippers as well as an open-source initiative for the dissemination of the dexterity test designs. The various evaluation methods proposed in the study have been validated using human trials. The output of these trials has been used to quantify dexterity based on the scoring methodology proposed. The importance of tactile feedback in performing these evaluations is also examined by performing the tasks with a padded glove and the results are presented. From the results, it is clearly evident that the task completion time decreases with trials for both set of experiments, indicating that a clear learning curve exists and that humans perform better after practising. The subjects took significantly longer to complete the tasks with the padded gloves. This clearly shows the importance of tactile feedback in performing dexterous manipulation. It is also clear from the robot gripper experiments that the human hands can complete the tasks in a very small fraction of the time taken by the robotic grippers indicating that there is a huge room for improvement of robotic devices.

## Data Availability

The raw data supporting the conclusions of this article will be made available by the authors, without undue reservation. The detailed evaluation protocol with explanatory images and scoring sheets, as well as the open-source CAD files of the proposed dexterity test are provided and can be downloaded from the following website: http://www.newdexterity.org/dexteritytest
